# Impact of Membranous Nectin-4 on Outcomes of Platinum-Based Chemotherapy in Metastatic Urothelial Carcinoma

**DOI:** 10.3390/cancers17030433

**Published:** 2025-01-27

**Authors:** Fu-Jen Hsueh, Chung-Chieh Wang, Jhe-Cyuan Guo, Shih-Chieh Chueh, Yu-Chieh Tsai

**Affiliations:** 1 Department of Oncology, National Taiwan University Hospital, Taipei 100, Taiwan; 017052@ntuh.gov.tw; 2 Department of Pathology, National Taiwan University Hospital, Taipei 100, Taiwan; wangchungchieh@ntuh.gov.tw; 3 Department of Medical Oncology, National Taiwan University Cancer Center, Taipei 106, Taiwan; A00524@ntucc.edu.tw; 4 Department of Urology, National Taiwan University Hospital, Taipei 100, Taiwan; chuehs@ntuh.gov.tw

**Keywords:** membranous nectin-4, platinum-based chemotherapy, metastatic urothelial carcinoma

## Abstract

**Background:** Platinum-based chemotherapy (Plt-ChT) remains a cornerstone treatment for metastatic urothelial carcinoma (mUC). The nectin-4-targeting antibody–drug conjugate enfortumab vedotin (EV), in combination with immune checkpoint inhibitors, has expanded first-line treatment options. However, biomarkers to guide treatment selection are lacking. Membranous nectin-4 (mNectin-4) expression, a potential marker of EV efficacy, has not been fully explored in the context of Plt-ChT. **Methods:** We retrospectively analyzed 96 patients with mUC treated with first-line gemcitabine plus cisplatin or carboplatin (GC/GCarbo) and classified mNectin-4 expression using immunohistochemical staining as either high (histochemical score (H-score): 100–300, mNectin-4^High^) or low (H-score: 0–99, mNectin-4^Low^). **Results:** Among these patients, 54.1% exhibited mNectin-4^High^ tumors, which were associated with bladder-origin tumors, a pure urothelial carcinoma histology, de novo presentation, and lymph node-only metastasis. The mNectin-4^High^ subgroup showed a non-significant trend toward better outcomes, including response rate (46.1% vs. 36.3%; *p* = 0.32), median progression-free survival (7.0 months vs. 4.0 months; adjusted *p* = 0.06), and median overall survival (20.0 months vs. 8.8 months; adjusted *p* = 0.06), compared with the mNectin-4^Low^ subgroup. The subgroup analysis revealed that the favorable outcomes for mNectin-4^High^ tumors were predominantly observed in the GC cohort, which was not evident in the GCarbo cohort. **Conclusions:** Our study suggests a non-significant trend toward improved outcomes with first-line Plt-ChT in patients with mNectin-4^High^ tumors, particularly with the GC regimen. Larger prospective studies are warranted to validate mNectin-4 as a potential predictive biomarker for mUC treatment.

## Abstract (plain language summary)

Metastatic urothelial carcinoma (mUC) is commonly treated with platinum-based chemotherapy (Plt-ChT) as the first-line regimen, but patient responses vary significantly, and predictive biomarkers are lacking. Nectin-4, a cell adhesion molecule and the target of the antibody–drug conjugate enfortumab vedotin, holds potential as a biomarker but has not been thoroughly investigated in the context of chemotherapy. This study evaluated whether membranous nectin-4 expression (mNectin-4) could predict the efficacy of Plt-ChT, including gemcitabine plus cisplatin or carboplatin, in mUC. Analyzing tumor samples from 96 patients revealed that higher mNectin-4 expression was associated with a non-significant trend towards improved outcomes, particularly with gemcitabine and cisplatin therapy. These findings suggest that mNectin-4 may serve as a potential predictive biomarker for refining treatment selection and personalizing therapeutic strategies for mUC. Further validation through larger prospective studies is required to confirm its clinical utility.

## 1. Introduction

Metastatic urothelial carcinoma (mUC) is a highly aggressive malignancy. The median overall survival (OS) after standard first-line platinum-based chemotherapy (Plt-ChT) is only 8–15 months, with a five-year survival rate of approximately 10% [1,2,3]. Recent advances, such as anti-programmed death-1/programmed death-ligand 1 immune checkpoint inhibitors (anti-PD-1/PD-L1 ICIs) and antibody–drug conjugates (ADCs), have revolutionized the first-line treatment of mUC. For instance, the phase III JAVELIN Bladder 100 trial confirmed that maintenance avelumab improved OS in patients without progression after initial gemcitabine plus cisplatin or carboplatin (GC/GCarbo) treatment [4]. Similarly, the phase III CheckMate 901 trial reported that adding nivolumab to GC enhanced OS in cisplatin-eligible patients [5]. Moreover, the phase III EV-302 study established the combination of enfortumab vedotin (EV, a nectin-4-targeting ADC) and pembrolizumab as a first-line option (the EV-P regimen), demonstrating significant survival benefits irrespective of cisplatin eligibility [6]. While first-line EV-P has shown unprecedented efficacy compared with other Plt-ChT-based regimens for mUC, chemotherapy remains a cornerstone treatment due to its affordability and accessibility, particularly in resource-limited settings. Furthermore, EV-P is often associated with substantial toxicities (e.g., skin reactions and neuropathy) and significant economic challenges [7]. Consequently, identifying biomarkers in mUC is essential for optimizing outcomes while reducing drug-related toxicities and mitigating financial burdens.

Nectin-4, a cell adhesion molecule, plays a critical role in tumor proliferation, migration, and invasion in many cancers—processes often associated with a poor prognosis [8,9,10,11,12,13,14]. It is highly expressed in urothelial carcinoma and has garnered attention as a potential biomarker [15,16]. In the exploratory analysis of the EV-302 study, researchers investigated the relationship between nectin-4 expression via immunohistochemistry (IHC) assay and oncological outcomes. They found that EV-P outperformed GC/GCarbo in all survival outcomes, irrespective of nectin-4 expression status [17]. However, the analysis did not specify whether nectin-4 immunoactivity was localized on the cell membrane or in the cytoplasm. Since nectin-4 is essential for EV binding [18], the membranous staining of nectin-4 (mNectin-4) is speculated to be a more appropriate biomarker than non-specified immunoactivity. Although the current evidence suggests that mNectin-4 expression is associated with EV effectiveness in patients with mUC [19], its prognostic and predictive role in the context of Plt-ChT remains unexplored.

To address these gaps, we conducted a retrospective study to assess the impact of mNectin-4 on the outcomes of first-line GC/GCarbo in patients with mUC. Additionally, we explored whether mNectin-4 expression affects the efficacy of cisplatin versus carboplatin-based regimens. This study aims to provide insights into optimizing treatment strategies for mUC, particularly in resource-limited settings where chemotherapy remains the primary therapeutic option.

## 2. Materials and Methods

### 2.1. Patient Cohort

We retrospectively enrolled histologically confirmed patients with mUC who received first-line GC/GCarbo treatment between 2012 and 2019 at the National Taiwan University Hospital (NTUH). During this period, patients could also receive ICIs following upfront chemotherapy, in accordance with international practice guidelines. We collected data on the clinical characteristics and treatment outcomes, including age, performance status, primary tumor locations, metastatic sites, objective response rate (ORR) as per the Response Evaluation Criteria in Solid Tumors (RECIST, version 1.1), progression-free survival (PFS), and OS. The final update of PFS and OS was conducted in November 2022. The study was approved by the Institutional Review Board of NTUH (approval numbers: 201912011RINC) and was performed in accordance with the Declaration of Helsinki.

### 2.2. Tissue Collection and IHC

Archival tumor tissues from primary sites, obtained before the initiation of first-line chemotherapy, were utilized for IHC analysis. For each case, a 5 μm section was taken from the formalin-fixed, paraffin-embedded tissue block. Staining procedures were conducted with a Ventana Benchmark ULTRA autostainer (Roche Diagnostics, Indianapolis, IN, USA) according to the manufacturer’s instructions. Following antigen retrieval with Discovery CC1 Solution (Roche Diagnostics, Indianapolis, IN, USA) for 64 min, the tissue slides were incubated with monoclonal anti-nectin-4 primary antibody (clone EPR15613-68, Abcam (Cambridge, UK), dilution 1:100) for 32 min at room temperature, with its specificity validated in the previous literature [19]. Reactivity was visualized using a Ventana OptiView DAB IHC Detection Kit (Roche Diagnostics, Indianapolis, IN, USA). Nectin-4 expression was evaluated under a light microscope by the same expreienced uro-pathologist (CCW). The IHC slides were then scanned using a Hamamatsu NanoZoomer S360 (Hamamatsu Photonics, Hamamatsu City, Japan), and the images were acquired with the AetherSlide Digital Pathology System (AetherAI, Taipei, Taiwan).

### 2.3. Semiquantitative Analysis of mNectin-4 Expression

Specific nectin-4 immunoreactivity in the cell membrane was assessed using the histochemical score system (H-score), which is calculated by summing the products of staining intensity (score: 0–3) and the percentage of stained cells (0–100) at each intensity level. The mNectin-4 expression levels were classified as strong (H-score: 200–300), moderate (H-score: 100–199), weak (H-score: 15–99), and negative (H-score: 0–14), as described in previous studies [16,20]. Strong and moderate staining of mNectin-4 were defined as high expression (mNectin-4^High^), while weak and negative staining were defined as low expression (mNectin-4^Low^).

### 2.4. Statistical Analysis

Statistical analyses were performed with MedCalc^®^ Statistical Software version 22.016 (MedCalc Software Ltd., Ostend, Belgium). An independent samples t-test and Fisher’s exact test were applied to compare the baseline characteristics of both subgroups. Median PFS and OS were estimated using the Kaplan–Meier method, and the survival differences between subgroups were assessed by a log-rank test. A Cox proportional hazards regression model was utilized to identify the independent predictors of survival. All *p* values were calculated as two-sided, and *p* < 0.05 was considered statistically significant.

## 3. Results

### 3.1. Patient Characteristics

A total of 96 patients with mUC were enrolled in this study. As of the data cutoff date (30 November 2022), the median follow-up time was 85.5 months. As shown in Table 1, the median age of the overall population was 64 years (range: 22–89), with most having an Eastern Cooperative Oncology Group (ECOG) performance score of 0–1. Approximately half had de novo metastases and visceral organ involvement. Most patients had a pure urothelial carcinoma histology (83.3%) and received first-line GC (80.2%). Only 19.8% received subsequent ICIs. Notably, up to 61.5% of patients in our cohort had upper tract urothelial carcinoma (UTUC). When examining characteristics stratified by mNectin-4 expression levels, patients with mNectin-4^High^ tumors were significantly more likely to have bladder-origin tumors, a pure urothelial carcinoma histology, de novo presentation, and lymph node (LN)-only metastasis compared with those with mNectin-4^Low^ tumors. Further demographic and clinical features are detailed in Table 1.

### 3.2. Expression of mNectin-4

**Figure 1:**
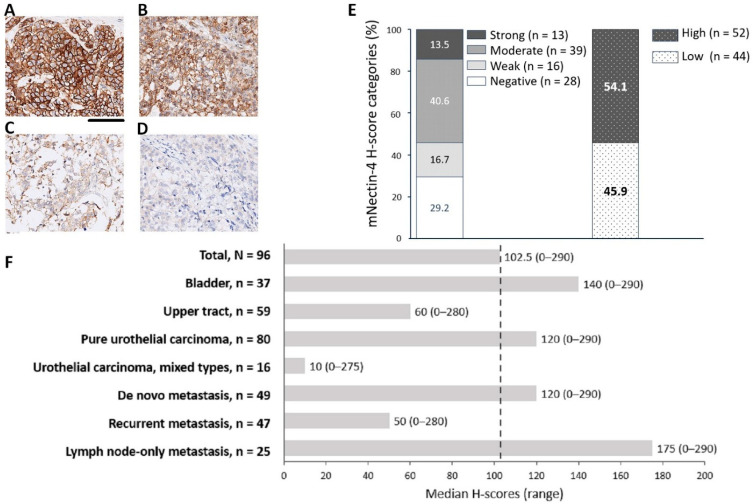
Membranous nectin-4 (mNectin-4) expression patterns in the total cohort and median histochemical score (H-scores) in selected subgroups. (**A**–**D**) Representative images of immunohistochemistry staining for strong (**A**), moderate (**B**), weak (**C**), and negative (**D**) mNectin-4 expression; scale bar: 100 μm. (**E**) Percentages of mNectin-4 expression categories. Strong and moderate staining were categorized as high expression (mNectin-4^High^), while weak and negative staining were categorized as low expression (mNectin-4^Low^). (**F**) Median H-scores in selected subgroups.

The expression pattern of mNectin-4 was classified as strong, moderate, weak, and negative, as defined earlier (Figure 1A–D). The proportions of patients with strong, moderate, weak, and negative mNectin-4 expression were 13.5%, 40.6%, 16.7%, and 29.2%, respectively. Overall, a total of 54.1% of patients had mNectin-4^High^ tumors (Figure 1E).

The median H-score for mNectin-4 in the total cohort was 102.5 (range: 0–290), with a median of 160 (range: 0–290) in the mNectin-4^High^ subgroup and 5 (range: 0–90) in the mNectin-4^Low^ subgroup. We further analyzed the median H-score in subgroups with significant differences in mNectin-4 expression (Figure 1F). Patients with LN-only metastasis had the highest median H-score (175), while those with mixed-type urothelial carcinoma had the lowest (10).

### 3.3. Oncological Outcomes for First-Line Chemotherapy Stratified by mNectin-4 Expression Levels

**Figure 2:**
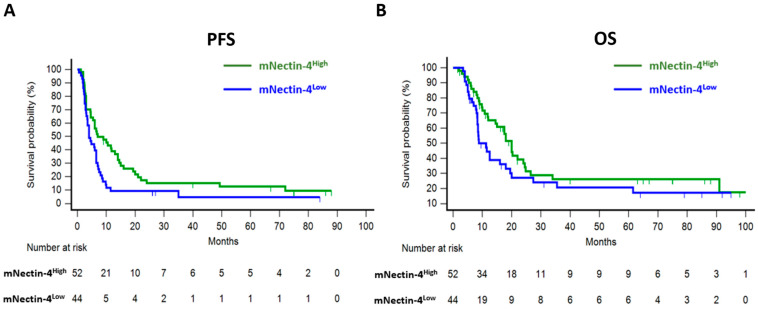
Survival analysis for first-line chemotherapy based on membranous nectin-4 (mNectin-4) expression status. Kaplan–Meier estimates of progression-free survival (PFS) (**A**) and overall survival (OS) (**B**).

Table 2 summarizes treatment outcomes for first-line chemotherapy. The ORR showed no significant difference between the mNectin-4^High^ and mNectin-4^Low^ subgroups (46.1% vs. 36.3%, *p* = 0.32). The median PFS was 6.0 months (95% confidence interval (CI): 4.0–7.0) overall, with 7.0 months (95% CI: 4.6–14.0) in the mNectin-4^High^ subgroup and 4.0 months (95% CI: 3.1–6.5) in the mNectin-4^Low^ subgroup (Figure 2A). Univariate analysis revealed that high mNectin-4 expression and bladder-origin tumors were associated with longer PFS, but these associations were not significant in the multivariable analysis (*p* = 0.06 and 0.07, respectively; Table 3). The median OS was 16.0 months (95% CI: 10.8–20.0) overall, with 20.0 months (95% CI: 14.5–24.5) in the mNectin-4^High^ subgroup and 8.8 months (95% CI: 8.3–18.0) in the mNectin-4^Low^ subgroup (Figure 2B). Both univariate and multivariable analyses identified an ECOG performance score > 0 as the only factor significantly associated with an inferior OS (Table 4).

### 3.4. Oncological Outcomes of the GC and GCarbo Cohorts Stratified by mNectin-4 Expression Levels

**Figure 3:**
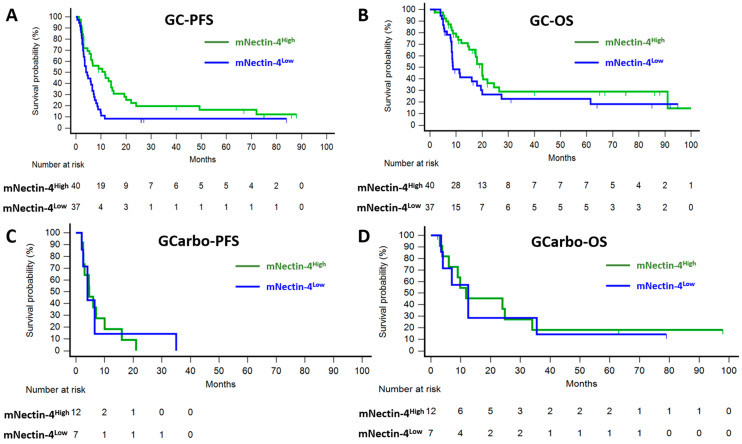
Survival analysis of the gemcitabine plus cisplatin (GC) and gemcitabine plus carboplatin (GCarbo) cohorts based on mNectin-4 expression status. Kaplan–Meier estimates of PFS (**A**) and OS (**B**) in the GC cohort. (**C**) PFS and (**D**) OS in the GCarbo cohort.

Next, we examined the impact of mNectin-4 on prognosis across different chemotherapy regimens. Table 5 summarizes the treatment outcomes of the GC and GCarbo cohorts based on mNectin-4 status. In the GC cohort, patients with mNectin-4^High^ tumors demonstrated a significantly longer PFS compared with those with mNectin-4^Low^ tumors (11.6 vs. 4.5 months; hazard ratio (HR): 0.48; 95% CI: 0.29–0.82; *p* = 0.007) (Figure 3A). A favorable trend was also observed for ORR (52.5% vs. 35.1%, *p* = 0.12) and OS (20.0 vs. 8.8 months; HR: 0.62; 95% CI: 0.35–1.10; *p* = 0.10) (Figure 3B). In the multivariable analysis, high mNectin-4 expression was associated with an improved PFS (HR: 0.62; 95% CI: 0.36–1.07; *p* = 0.08) (Table 6) and OS (HR: 0.56; 95% CI: 0.31–1.04; *p* = 0.07) (Table 7) in the GC cohort. Conversely, no favorable trends were observed for mNectin-4^High^ tumors in the GCarbo cohort across ORR, PFS (Figure 3C), or OS (Figure 3D).

## 4. Discussion

Compared with studies on EV, the role of nectin-4 in chemotherapy for mUC remains underexplored. For instance, one study examining *NECTIN-4* copy number variations in 103 patients with mUC treated with palliative Plt-ChT, with or without ICI combinations, found no significant association with OS [21]. Similarly, another study reported no correlation between *NECTIN-4* RNA expression and OS in patients with mUC receiving platinum-based therapies [22]. Consistent with these findings, our study did not identify a significant difference in OS between mNectin-4^High^ and mNectin-4^Low^ tumors following first-line Plt-ChT, although a favorable trend was observed in the mNectin-4^High^ subgroup. Notably, our research displays several key strengths compared with prior studies. First, nectin-4 expression was assessed using IHC, a method more practical and clinically accessible than genomic analyses. Second, we specifically focused upon nectin-4 immunoactivity on the cell membrane, a biomarker more relevant for evaluating the efficacy of EV- versus Plt-ChT-based regimens in first-line treatment. Third, we analyzed a homogeneous cohort of patients uniformly treated with first-line GC or GCarbo regimens, aligning with the control arms of the JAVELIN Bladder 100 and CheckMate 901 trials and reflecting real-world clinical practice. Lastly, we adjusted OS for several prognostic factors, including ECOG performance status, first-line GC use, and subsequent ICI administration, to provide a more comprehensive evaluation of mNectin-4’s impact on Plt-ChT efficacy in mUC.

While our study primarily investigates the role of mNectin-4 in the context of first-line Plt-ChT, the evolving landscape of systemic treatments for later-line mUC, including ICIs and ADCs, warrants consideration [23,24,25,26]. In our cohort, up to 20% of patients received subsequent ICIs, which may have influenced survival outcomes. Currently, studies examining the impact of nectin-4 on ICI responsiveness in mUC are limited. However, one study reported that strong nectin-4 expression was associated with a higher disease control rate for pembrolizumab following chemotherapy [27]. This highlights the need for future biomarker research, including mNectin-4, to explore its role in the efficacy of novel agents in later-line treatments.

Intriguingly, we observed a favorable trend in PFS and OS for mNectin-4^High^ tumors in the GC cohort, a pattern not observed in the GCarbo cohort. The underlying mechanisms of these findings remain elusive but likely reflect the interplay between tumor biology and the immunomodulatory properties of different chemotherapeutic agents. For instance, *NECTIN-4* gene expression demonstrates heterogeneity across molecular subtypes of bladder cancer and is notably enriched in luminal subtypes [18]. Moreover, elevated *NECTIN-4* RNA expression correlates with a high tumor mutation burden, reduced PD-L1 expression, and lower immune cell fractions [22]. Notably, the GC regimen has been shown to upregulate immune-related genes in the circulating monocytes involved in antigen presentation and T cell priming compared with the GCarbo regimen [28]. This difference may help to explain the divergent outcomes observed between the two cohorts. Given the encouraging trends associated with the GC regimen, mNectin-4 may represent a promising biomarker, particularly in cisplatin-eligible patients with mUC. Future investigations should further delineate the distinct roles of mNectin-4 expression in cisplatin- versus carboplatin-based regimens, ideally integrating molecular profiling to elucidate the underlying mechanisms.

Our study has several limitations. First, its retrospective design introduces inherent selection bias. Second, the high prevalence of UTUC in our cohort is notable. Because UTUC exhibits distinct transcriptomic profiles and clinical outcomes [29,30], our findings may have limited generalizability to patients with bladder urothelial carcinoma. Nevertheless, the inclusion of UTUC provides valuable insights, particularly in regions with high incidence rates [31]. Third, a significantly higher proportion of patients in our cohort received GC compared with GCarbo, which may have influenced survival outcomes and subsequent analyses. This imbalance likely reflects clinicians’ preference for administering more effective treatments, even when using split-dosing schedules for GC. Finally, the relatively small sample size limits the statistical power and generalizability of our conclusions. To validate these findings and confirm the utility of mNectin-4 as a predictor for mUC, future research should prioritize prospective studies with larger, multicenter cohorts to minimize selection bias and improve generalizability. Additionally, stratifying patients by tumor location and balancing treatment regimens will be crucial for understanding the role of mNectin-4 in optimizing therapeutic approaches for mUC.

## 5. Conclusions

In summary, our study provides real-world evidence suggesting a non-significant trend toward favorable outcomes for patients with mNectin-4^High^ tumors treated with first-line Plt-ChT, particularly with the GC regimen. These findings highlight the potential of mNectin-4 in guiding treatment decisions in mUC. Beyond chemotherapy, future research may merit further exploration into the broader applications of mNectin-4, including its role in ICIs and ADCs, to better personalize and refine therapeutic strategies for mUC.

## Figures and Tables

**Table 1:** Demographic and clinical features of the study population.

Characteristic	Total (n = 96)	mNectin-4^High^ (n = 52)	mNectin-4^Low^ (n = 44)	*p* Value
Median age, years (range)	64 (22–89)	63 (22–85)	65 (43–89)	0.95
Gender, n (%)				0.82
Male	47 (49)	26 (50.0)	21 (47.7)	
Female	49 (51)	26 (50.0)	23 (52.3)	
ECOG performance score, n (%)				
0	28 (29.2)	13 (25.0)	15 (34.1)	0.33
1	64 (66.7)	36 (69.2)	28 (63.6)	0.56
2	4 (4.1)	3 (5.8)	1 (2.3)	0.39
Number of Bajorin risk factors, n (%)				0.92
0	42 (43.7)	23 (44.2)	19 (43.2)	
1	54 (56.3)	29 (55.8)	25 (56.8)	
Median H-score of mNectin-4 expression (range)	102.5 (0–290)	160 (100–290)	5 (0–90)	<0.0001 ***
Pattern of metastasis, n (%)				0.008 **
De novo	49 (51)	33 (63.5)	16 (36.4)	
Recurrent	47 (49)	19 (36.5)	28 (63.6)	
Primary site of disease, n (%)				0.03 *
Upper tract †	59 (61.5)	27 (51.9)	32 (72.7)	
Bladder	37 (38.5)	25 (48.1)	12 (27.3)	
Histology type, n (%)				0.002 **
Urothelial carcinoma, pure	80 (83.3)	49 (94.2)	31 (70.5)	
Urothelial carcinoma, mixed types §	16 (16.7)	3 (5.8)	13 (29.5)	
Sites of metastasis, n (%)				
Liver	16 (16.7)	7 (13.5)	9 (20.5)	0.36
Visceral organs	54 (56.3)	29 (55.8)	25 (56.8)	0.92
LN only	25 (26)	20 (38.5)	5 (11.4)	0.002 **
First-line chemotherapy regimen, n (%)				0.38
GC	77 (80.2)	40 (76.9)	37 (84.1)	
GCarbo	19 (19.8)	12 (23.1)	7 (15.9)	
Subsequent ICIs, n (%)				0.71
Yes	19 (19.8)	11 (21.2)	8 (18.2)	
No	77 (80.2)	41 (78.8)	36 (81.8)	

Abbreviations: ECOG: Eastern Cooperative Oncology Group; GC: gemcitabine plus cisplatin; GCarbo: gemcitabine plus carboplatin; H-score: histochemical score; ICIs: immune checkpoint inhibitors; LN: lymph node; mNectin-4: membranous nectin-4. * *p* < 0.05, ** *p* < 0.01, *** *p* < 0.001, statistically significant. Bajorin risk factors include visceral metastases (metastases to the lung, bone, or liver) and an ECOG performance status score of 3 or higher. † Upper tract primary sites include the renal pelvis and ureter. § This category includes squamous, sarcomatoid, clear cell, and partial nested features.

**Table 2:** Oncological outcomes of first-line chemotherapy stratified by membranous nectin-4 (mNectin-4) expression levels (total cohort, n = 96).

Outcomes	Total (n = 96)	mNectin-4^High^ (n = 52)	mNectin-4^Low^ (n = 44)	HR (95% CI)	*p* Value
ORR, n (%)	40 (41.6)	24 (46.1)	16 (36.3)		0.32
PFS, months (95% CI)	6.0 (4.0–7.0)	7.0 (4.6–14.0)	4.0 (3.1–6.5)	0.55 (0.35–0.88)	0.01 *
OS, months (95% CI)	16.0 (10.8–20.0)	20.0 (14.5–24.5)	8.8 (8.3–18.0)	0.66 (0.40–1.10)	0.11

Abbreviations: CI: confidence interval; HR: hazard ratio; mNectin-4: membranous nectin-4; ORR: objective response rate; OS: overall survival; PFS: progression-free survival. * *p* < 0.05, statistically significant.

**Table 3:** Cox regression analysis of progression-free survival (PFS) in the total cohort (n = 96).

Variable	PFS			
	Univariate		Multivariable	
	HR (95% CI)	*p* Value	HR (95% CI)	*p* Value
mNectin-4 expression (high vs. low)	0.55 (0.35–0.88)	0.01 *	0.65 (0.41–1.02)	0.06
ECOG (≥1 vs. 0)	1.47 (0.92–2.35)	0.10		
Primary site (bladder vs. upper tract)	0.59 (0.38–0.92)	0.02 *	0.64 (0.40–1.04)	0.07
Visceral metastasis (yes vs. no)	1.14 (0.73–1.77)	0.56		
LN-only metastasis (yes vs. no)	0.81 (0.50–1.31)	0.39		
Histology (mixed vs. pure UC)	1.69 (0.88–3.23)	0.11		
First-line GC (yes vs. no)	0.66 (0.36–1.19)	0.17		
Subsequent ICIs (yes vs. no)	1.32 (0.75–2.34)	0.32		

Abbreviations: CI: confidence interval; ECOG: Eastern Cooperative Oncology Group; GC: gemcitabine plus cisplatin; HR: hazard ratio; ICIs: immune checkpoint inhibitors; LN: lymph node; mNectin-4: membranous nectin-4; PFS: progression-free survival; UC: urothelial carcinoma. * *p* < 0.05, statistically significant.

**Table 4:** Cox regression analysis of overall survival (OS) in the total cohort (n = 96).

Variable	OS			
	Univariate		Multivariable	
	HR (95% CI)	*p* Value	HR (95% CI)	*p* Value
mNectin-4 expression (high vs. low)	0.66 (0.40–1.10)	0.11	0.62 (0.37–1.03)	0.06
ECOG (≥1 vs. 0)	1.88 (1.12–3.16)	0.01 *	2.56 (1.36–4.83)	0.003 **
Primary site (bladder vs. upper tract)	0.65 (0.40–1.07)	0.09		
Visceral metastasis (yes vs. no)	1.41 (0.87–2.30)	0.09		
LN-only metastasis (yes vs. no)	0.66 (0.39–1.12)	0.12		
Histology (mixed vs. pure UC)	1.15 (0.56–2.37)	0.68		
First-line GC (yes vs. no)	0.81 (0.44–1.51)	0.52		
Subsequent ICIs (yes vs. no)	0.79 (0.45–1.37)	0.40		

* *p* < 0.05, ** *p* < 0.01, statistically significant.

**Table 5:** Oncological outcomes of the gemcitabine plus cisplatin (GC) and gemcitabine plus carboplatin (GCarbo) cohorts stratified by mNectin-4 expression levels.

Outcomes	GC (n = 77)				GCarbo (n = 19)			
	mNectin-4^High^ (n = 40)	mNectin-4^Low^ (n = 37)	HR (95% CI)	*p* Value	mNectin-4^High^ (n = 12)	mNectin-4^Low^ (n = 7)	HR (95% CI)	*p* Value
ORR, n (%)	21 (52.5)	13 (35.1)		0.12	3 (25.0)	3 (42.8)		0.43
PFS, months (95% CI)	11.6 (5.4–14.5)	4.5 (3.0–6.5)	0.48 (0.29–0.82)	0.007 **	4.6 (2.50–10.0)	4.0 (2.0–6.5)	1.06 (0.38–2.94)	0.91
OS, months (95% CI)	20.0 (15.0–24.5)	8.8 (8.3–19.5)	0.62 (0.35–1.10)	0.10	11.8 (3.8–34.0)	12.5 (3.4–35.5)	0.95 (0.33–2.72)	0.92

** *p* < 0.01, statistically significant.

**Table 6:** Cox regression analysis for PFS in the GC cohort (n = 77).

Variable	PFS			
	Univariate		Multivariable	
	HR (95% CI)	*p* Value	HR (95% CI)	*p* Value
mNectin-4 expression (high vs. low)	0.48 (0.29–0.82)	0.007 **	0.62 (0.36–1.07)	0.08
ECOG (≥1 vs. 0)	1.52 (0.91–2.54)	0.10		
Primary site (bladder vs. upper tract)	0.61 (0.37–1.00)	0.05		
Visceral metastasis (yes vs. no)	1.22 (0.74–2.00)	0.56		
LN-only metastasis (yes vs. no)	0.76 (0.45–1.28)	0.30		
Histology (mixed vs. pure UC)	2.27 (1.10–4.67)	0.02 *	1.45 (0.77–2.72)	0.25
Subsequent ICIs (yes vs. no)	1.60 (0.77 –3.35)	0.21		

* *p* < 0.05, ** *p* < 0.01, statistically significant.

**Table 7:** Cox regression analysis of OS in the GC cohort (n = 77).

Variable	OS			
	Univariate		Multivariable	
	HR (95% CI)	*p* Value	HR (95% CI)	*p* Value
mNectin-4 expression (high vs. low)	0.62 (0.35–1.10)	0.10	0.56 (0.31–1.04)	0.07
ECOG (≥1 vs. 0)	1.87 (1.05–3.33)	0.03 *	2.60 (1.29–5.22)	0.007 **
Primary site (bladder vs. upper tract)	0.58 (0.33–1.01)	0.06		
Visceral metastasis (yes vs. no)	1.48 (0.85–2.59)	0.17		
LN-only metastasis (yes vs. no)	0.67 (0.37–1.20)	0.18		
Histology (mixed vs. pure UC)	1.30 (0.58–2.88)	0.52		
Subsequent ICIs (yes vs. no)	0.93 (0.47–1.86)	0.84		

* *p* < 0.05, ** *p* < 0.01, statistically significant.

## Data Availability

All data generated or analyzed during this study are included in the published article.
